# Time matters: The prognostic impact of diagnostic delay on survival in primary central nervous system lymphoma—a single-center, retrospective real-world study

**DOI:** 10.1093/noajnl/vdaf234

**Published:** 2025-10-27

**Authors:** Louisa Lehner, Louisa von Baumgarten, Jonas Reis, Aamna Khan, Kim-Fabienne Degmayr, Benjamin Englert, Andreas Straube, Martin Dreyling, Veit Stöcklein, Patrick N Harter, Niklas Thon, Stefanie Quach, Katharina J Müller

**Affiliations:** Department of Neurology, LMU University Hospital, LMU Munich, Munich (L.L., L.V.B., A.S., K.J.M.); Department of Neurology, LMU University Hospital, LMU Munich, Munich (L.L., L.V.B., A.S., K.J.M.); Department of Neurosurgery, LMU University Hospital, LMU Munich, Munich (L.V.B., A.K., V.S., K.-F.D., V.S., N.T., S.Q.); Department of Medicine III, LMU University Hospital of Munich, LMU Munich, Munich (L.V.B., M.D.); Department of Neuroradiology, LMU University Hospital, LMU Munich, Munich (J.R.); Department of Neurosurgery, LMU University Hospital, LMU Munich, Munich (L.V.B., A.K., V.S., K.-F.D., V.S., N.T., S.Q.); Department of Neurosurgery, LMU University Hospital, LMU Munich, Munich (L.V.B., A.K., V.S., K.-F.D., V.S., N.T., S.Q.); Center for Neuropathology and Prion Research, Faculty of Medicine, LMU Munich, Munich (B.E., P.N.H.); Department of Neurology, LMU University Hospital, LMU Munich, Munich (L.L., L.V.B., A.S., K.J.M.); Department of Medicine III, LMU University Hospital of Munich, LMU Munich, Munich (L.V.B., M.D.); Department of Neurosurgery, LMU University Hospital, LMU Munich, Munich (L.V.B., A.K., V.S., K.-F.D., V.S., N.T., S.Q.); German Cancer Consortium (DKTK), Partner Site Munich, German Cancer Research Center (DKFZ), Heidelberg (V.S., P.N.H.); Center for Neuropathology and Prion Research, Faculty of Medicine, LMU Munich, Munich (B.E., P.N.H.); German Cancer Consortium (DKTK), Partner Site Munich, German Cancer Research Center (DKFZ), Heidelberg (V.S., P.N.H.); Department of Neurosurgery, LMU University Hospital, LMU Munich, Munich (L.V.B., A.K., V.S., K.-F.D., V.S., N.T., S.Q.); Department of Neurosurgery, LMU University Hospital, LMU Munich, Munich (L.V.B., A.K., V.S., K.-F.D., V.S., N.T., S.Q.); Department of Neurology, LMU University Hospital, LMU Munich, Munich (L.L., L.V.B., A.S., K.J.M.)

**Keywords:** diagnostic delay, PCNSL, primary CNS lymphoma, prognostic factors

## Abstract

**Abstract:**

BackgroundPrimary central nervous system lymphoma (PCNSL) is a rare and aggressive malignancy that frequently mimics other central nervous system (CNS) diseases, leading to diagnostic delays. Given its often nonspecific radiological presentation, PCNSL remains a diagnostic challenge, however early diagnosis and timely initiation of treatment are critical. This study aimed to evaluate diagnostic timelines and their influencing factors, treatment patterns, and their impact on survival in patients with PCNSL.

**Methods:**

We retrospectively analyzed 125 patients diagnosed with PCNSL at a single tertiary care referral center between 2008 and 2021. Clinical, radiological, and histopathological data were collected to assess factors influencing diagnostic delay, treatment decisions, and patient outcomes.

**Results:**

The median age at diagnosis was 68 years (21-89) and median Karnofsky Performance Status (KPS) was 70% (10-100). The median time from initial clinical symptom to histopathologically confirmed diagnosis was 37 days (4-749). The median time from first neuroimaging to confirmed diagnosis was 12 days (2-225). A shorter diagnostic interval (≤ 12 days) was associated with significantly improved overall survival and progression-free survival (PFS) (*P* < .05). In a multivariate Cox proportional hazards model, predictors of OS were KPS ≥70% (*P* < .003), preserved renal function (GFR > 60 mL/min, *P* < .027), and MTX-based chemotherapy (*P* < .001). Further, diagnostic delay (>12 days) emerged as an independent predictor of PFS (*P* < .024).

**Conclusion:**

Our study underscores the prognostic impact of diagnostic delay in PCNSL. Renal function and KPS emerged as independent OS markers. MTX-based chemotherapy remains the standard of care, with autologous hematopoietic stem cell transplantation providing best survival outcomes in eligible patients.

Key Points

- A shorter diagnostic interval is associated with superior progression free survival.
- KPS, renal function and choice of treatment are the essential prognostic factors in PCNSL though.
- As diagnostic delay is modifiable, increasing clinical awareness and establishing standardized diagnostic protocols could significantly improve patient outcomes

Importance of the Study

PCNSL is a highly aggressive and rapidly progressing tumor, making early diagnosis and timely treatment initiation critical for improving patient outcomes. To our knowledge, this is the first study to identify diagnostic delay as an independent prognostic factor for progression-free survival (PFS) in PCNSL. In addition to diagnostic speed, key prognostic factors include the patient’s general condition (KPS), renal function, and treatment choice. Methotrexate-based chemotherapy, particularly when combined with autologous stem cell transplantation (ASCT), offers best survival outcomes and should be prioritized whenever feasible.

Unlike known prognostic factors such as KPS or renal function, diagnostic delay is modifiable, making its identification as a prognostic factor particularly relevant. Implementing standardized diagnostic protocols could accelerate the workup and facilitate earlier treatment initiation. Future research should focus on identifying specific causes of diagnostic delay to enable improvement in clinical practice.

Primary central nervous system lymphoma (PCNSL) is a rare and in most cases aggressive extranodal high-grade non-Hodgkin B-cell-lymphoma, manifesting in the brain, leptomeninges, spinal cord, or eyes.^1^ PCNSL are highly malignant and rapidly progressing and the disease course without treatment is typically fatal.^2^

It accounts for approximately 4% of all primary CNS tumors, with an estimated incidence of 0.5 per 100,000 individuals.^3^^,^^4^ PCNSL predominantly affects older adults and presents with a broad spectrum of nonspecific symptoms, including cognitive decline, personality changes, or focal neurological deficits, depending on tumor localization.^5^ The disease often exhibits an infiltrative growth pattern within deep brain structures and frequently presents with multifocal lesions.^6^^,^^7^ Differentiating PCNSL from other CNS pathologies, such as glioblastoma, brain metastases, or autoimmune disorders like small-vessel vasculitis, can be challenging based on neuroimaging alone.^8^ Histopathological confirmation, typically obtained via stereotactic biopsy or minimal-invasive frame-less biopsy procedures, remains the gold standard for diagnosis. However, the phenomenon of steroid responsiveness (described in nearly half of PCNSL patients) can obscure histopathological findings, leading to inconclusive biopsy results and potentially delaying definitive treatment initiation.^9^

Compared to systemic diffuse large B-cell lymphoma (DLBCL), PCNSL has a poorer prognosis.^2^ However, outcomes have significantly improved over the past two decades with a reported median overall survival (OS) of 25.3 months in recent real-world studies.^10^ Still, due to the rarity of PCNSL and the limited availability of randomized clinical trials, no universally accepted standard treatment exists: High-dose methotrexate (HD-MTX)-based chemotherapy remains the cornerstone of treatment and has significantly improved median OS in recent years. For younger, physically fit patients, consolidation with autologous hematopoietic stem cell transplantation (ASCT) has demonstrated superior efficacy and the potential for long-term remission or cure.^11^^,^^12^ Despite advances in PCNSL treatment, 75% do not respond long lasting to the initial treatment and it remains unclear if the delay of initiation of therapy influences the responder rate.^10^^,^^11^^,^^13^

Diagnostic delays have been associated with adverse outcomes in many cancers and are well-studied in solid tumors. However, despite the highly aggressive nature of PCNSL, data on the impact of diagnostic delay in this disease remains limited. Given the rapid progression of PCNSL, timely diagnosis and treatment initiation are of critical importance.

This study aimed to assess the temporal dynamics from symptom onset to diagnosis, evaluate treatment patterns, and investigate their impact on clinical outcomes in patients diagnosed with PCNSL at our institution.

## Methods

### Study Population

For this single-center, retrospective study, we identified patients with newly diagnosed histopathologically confirmed PCNSL, diagnosed between January 2008 and January 2021 at the University Hospital of Munich, Ludwig Maximilian’s University (LMU). The study was approved by the institutional ethics review board (No. 21-0320). Inclusion criteria were age ≥ 18 years at the time of diagnosis and a histopathological confirmed PCNSL. The data were extracted from medical records and anonymized for statistical analysis.

### Patient Characteristics

Demographic data, clinical features, neuroimaging and treatment regimens were assessed. Karnofsky performance status scale (KPS), Memorial Sloan-Kettering Cancer (MSKCC)^14^ and International Extra-nodal Lymphoma Study Group (IELSG) scores were calculated using patients’ baseline characteristics.^15^ The time of diagnosis was defined as the time of histopathological confirmed diagnosis of PCNSL. The date of the pathology report was assumed to be the date of diagnosis. We calculated the time span from symptom onset until histopathological confirmed diagnosis as well as the time from first neuroimaging to diagnosis. Additionally, time span from diagnosis until initiation of treatment was assessed. Routine MRI included gadolinium-enhanced T1-weighted, and nonenhanced T1- and T2-weighted sequences. According to the consensus recommendations by the International Primary CNS Lymphoma Collaborative Group (IPCG), tumor extension was delineated via the assessment of the sum of products of maximal cross-sectional diameters (SPD) of the contrast-enhancing lesion as well as of the nonenhancing components (Institutional programme: Visage Imaging, Inc., San Diego, California, United States).^16^

Therapy groups were defined as follows: MTX-based polychemotherapy with ASCT, MTX-based polychemotherapy without ASCT, non-MTX-based polychemotherapy without ASCT, radiotherapy and best supportive care. The primary outcomes were OS calculated from the time of diagnosis to time of death and PFS calculated from the time of diagnosis and MRI showing progression or recurrence.

### Statistical Analyses

Statistical analyses were carried out using SPSS Version 28 and GraphPad Prism 10 (GraphPad Software Inc., San Diego, California, United States). Categorical data are expressed as numbers (%), continuous values are expressed as median and range. Two group comparison was made using *t*-test, if normally distributed, for data not normally distributed nonparametric testing via Mann-Whitney *U* test was applied for comparison between two groups and the Kruskal-Wallis test for comparisons among multiple groups (with post-hoc Dunn′s testing). Statistical significance was assessed via the Cox proportional regression analysis. Variables demonstrating statistical significance or approaching significance in the univariate analysis were incorporated into a multivariate analysis employing the Cox proportional hazards regression model. Additionally, Kaplan-Meier estimator was used for survival curves, the log rank test was used to assess statistical significance. *P* < .05 were considered statistically significant.

## Results

### Patient Characteristics

Between 2008 and 2021, a total of 127 patients with newly diagnosed PCNSL were diagnosed or treated at our institution. Two patients were excluded due to missing data: one had an unclear medical history, and one diagnosed postmortem. Thus, 125 patients with PCNSL were included in the analysis. The baseline characteristics of these patients are summarized in Table 1. The median age at diagnosis was 68 years (21-89), and the median KPS was 70% (10-100). The most common initial symptoms were cognitive deficits (35.2%), followed by motor, sensory or visual deficits (30.4%), and cerebellar symptoms (21.6%). Epileptic seizures occurred in 8.8% of cases, while aphasia was the least common initial symptom (4.0%). The IELSG-Score could be calculated for 109 patients (87.2%); 27.2% had a score below three, while 60% had a score of three or higher. The MSKCC score was calculable in all patients, 13.6% had a 1 point, 53.6% had a score of 2 and 32.8% had a score of 3. Seven patients (5.6%) were HIV positive, and 12 patients (9.6%) were tested positive for EBV (Table 1).

**Table 1.** Demographic and clinical characteristics

Characteristics	*N* (%)/Median (range)
Sex	
Male	73 (58.4)
Female	52 (41.6)
Age	68 (21-89)
KPS	70 (10-100)
Leading symptom	
Cognitive deficits	44 (35.2)
Epileptic seizure	11 (8.8)
Focal neurological deficits (sensory, motor, visual)	38 (30.4)
Cerebellar symptoms	27 (21.6)
Aphasia	5 (4.0)
Diagnostic method	
Biopsy	116 (92.8)
Open tumour resection	6 (4.8)
CSF only	3 (2.4)
Re-Biopsy	6 (4, 8)
Ki67	80 (20-100)
HIV status: positive	
HIV positive	7 (5.6)
EBV association	12 (9, 6)
EBV association and HIV positive	5 (41, 6)
Permanent oral immunosuppressing medication	12 (9.6%)
No permanent oral immunosuppressing medication	113 (90.4%)
Drug immunosuppression 14 days prior to biopsy	
No immunosuppressants	77 (61.6)
Glucocorticoids	45 (36.0)
Other immunosuppressants	3 (2.4)
IELSG Score	
0	1 (0.8)
1	8 (6.4)
2	25 (20.0)
3	37 (29.6)
4	24 (19.2)
5	14 (11.2)
Not available	16 (12.8)
MSKCC Score	
1	17 (13.6)
2	67 (53.6)
3	41 (32.8)
CSF	
Atypical cells	21 (16.8)
Lympho-/mono-/granulocytosis	42 (33.6)
Normal cell count	50 (40.0)
Not available	12 (9.6)
Involvement of deep brain structures	92 (73.6)
No involvement of deep brain structures	33 (26.4)
Singular lesion	46 (36.8)
Multiple lesions	78 (62.4)
Not available	1 (0.8)
Tumor area (SPD) (cm^2^)	6.9 (0-45.5)
Localisation	
Supratentorial only	77 (61.6)
Infratentorial only	17 (13.6)
Supra- and infratentorial	28 (22.4)
Spinal	1 (0.8)
Not available	2 (1.6)
Ocular involvement	7 (5.6)
No ocular involvement	118 (94.4)
Initial radiological assessment	
CNS lymphoma	86 (68.8)
Other differential diagnoses	39 (31.2)
Creatinine clearance	
< 50	10
50-99	67
≥ 100	40
not available	8
Therapy regimen	
MTX-based chemotherapy	91 (72.8)
MTX-based chemotherapy with SCT	14 (11.2)
Other chemotherapy (non-MTX based)	7 (5.6)
Radiotherapy	11 (8.8)
No therapy	2 (1.6)
Therapy associated complications	108 (86.4)
No complications	13 (10.4)
Not available	6 (3.2)

Regarding immunosuppressive therapy, 12 patients (9.6%) were receiving long-term oral immunosuppressants, whereas 90.4% had no history of chronic immunosuppressive medication. However, in the 14 days preceding biopsy, 48 patients (38.4%) had received immunosuppressive treatment: 36% were taking glucocorticoids, and 2.4% had been treated with other immunosuppressants (e.g., azathioprine, mycophenolate mofetil).

In terms of geographic distribution, 35.2% of patients resided in metropolitan areas of Munich, close to a maximum care hospital, while 64.8% lived in rural areas, including different states or foreign countries (Table 1).

### Radiological and Histopathological Findings

Neuroimaging revealed deep brain structure involvement in 92 patients (73.6%). The majority of cases (61.6%) exhibited supratentorial localization, while one patient had an isolated spinal lymphoma. Ocular involvement was detected in seven patients (5.6%). Leptomeningeal disease, defined as the detection of atypical cells in the cerebrospinal fluid (CSF), was present in 21 patients (16.8%) (Table 1). Tumor area on CE-T1-weighted MRI revealed a median SPD of 6.9$$cm^2^. On initial MRI or CT scans, 68.8% of cases were correctly identified as lymphoma or probable lymphoma. In contrast, 31.2% were initially misdiagnosed, with alternative differential considerations such as inflammatory lesions, metastases, or infectious/autoimmune conditions (Supplementary Figure S1).

Histopathological confirmation was obtained in 116 patients (92.8%) through stereotactic biopsy, while six patients (4.8%) underwent open tumor resection. In three patients (2.4%), the diagnosis was established via histopathological CSF analysis. A second biopsy was required in six patients (4.8%), but in all cases, the second biopsy was performed due to suspected recurrence rather than inconclusive histopathological findings from the initial biopsy.

### Diagnostic and Therapeutic Delay

The median time from initial clinical symptom to histopathologically confirmed diagnosis was 37 days (4-749). The median interval between the first neuroimaging and confirmed histopathological diagnosis was 12 days (2-225). The median time from first neuroimaging until treatment initiation was 20 days (4-228), while the median time from histopathological confirmation to treatment initiation was 7 days (0-81). In one patient, chemotherapy was started based on the intraoperative histological analysis, defined as day zero.

Establishment of diagnosis and time from first neuroimaging until treatment initiation tended to be longer when the initial radiological assessment led to a misdiagnosis, suggesting an alternative condition instead of lymphoma (*P* = .055, *P* = .51; Table 2). Patients receiving immunosuppressive medication prior to biopsy experienced a statistically significant diagnostic delay and consequently a delayed initiation of treatment compared to those not receiving immunosuppressants in the 14 days preceding biopsy. This delay was evident in the time from initial symptom onset to histopathological confirmation (*P* = .04), in the time from first neuroimaging to histopathological diagnosis (*P* < .001) and in the time from first neuroimaging until treatment (*P* < .001). However, pre-biopsy immunosuppressive therapy did not significantly affect the time from diagnosis to treatment initiation.

**Table 2.** Time from initial clinical symptom/first neuroimaging to histopathological confirmed diagnosis and time from diagnosis to treatment according to demographical and clinical factors.

	Time from first neuroimaging until histopathological diagnosis		Time from initial symptom until ­histopathological diagnosis		Time from ­histopathological diagnosis to treatment		Time from first neuroimaging until treatment	
	Median (range)	*P*-value	Median (range)	*P*-value	Median (range)	*P*-value	Median (range)	*P*-value
Male	11 (3-110)	.121	37 (4-389)	.615	7 (0-31)	.425	19 (4-228)	.451
Female	14 (2-225)		36 (8-749)		6 (0-81)		22 (4-114)	
Age <65 years	12 (3-225)	.499	35 (4-749)	.558	6 (0-81)	.740	20 (4-228)	.925
Age ≥ 65 years	13 (2-110)		38 (6-506)		7 (0-31)		19 (4-114)	
KPS ≥ 70%	9,5 (2-225)	.061	35 (4-506)	.130	7 (0-81)	.293	20 (4-228)	.386
KPS < 70%	14 (3-78)		40 (6-749)		7 (0-18)		20 (4-82)	
Drug immunosuppression 14 days prior to biopsy	18 (2-225)	**< .001**	46 (10-749)	**.004**	8 (0-31)		25.5 (4-228)	<.001
No immunosuppressants	9 (2-110)		32 (4-506)		6 (0-81)	.074	16 (4-114)	
No involvement of deep brain structures	10 (2-110)	.415	26 (4-506)	.260	8 (1-81)	.143	21 (6-114)	.722
Involvement of deep brain structures	12 (2-225)		37 (6-749)		6.5 (0-31)		20 (4-228)	
Main symptom		.113		.248		.320		.264
Cognitive impairment	10 (3-77)		40 (8-179)		8 (0-18)		18.5 (8-82)	
Other neurological deficits	13 (2-225)		35 (4-749)		6 (-2-81)		23 (4-228)	
Metropolitan area	12 (2-225)	.981	35 (8-389)	.559	8 (0-31)	.104	20 (4-228)	.852
Rural area	12 (2-110)		37 (4-749)		6 (0-81)		20 (4-114)	
Initial radiological assessment		.055		.351		0.885	19 (4-114)	.051
CNS lymphoma	11 (2-110)		35 (6-749)		7 (0-31)		27 (4-228)	
Other differential diagnoses	19 (2-225)		40 (4-506)		7 (0-81)			

*P*-value was calculated using Mann-Whitney *U* test. Bold values indicate statistical significance (*P* < .05).

No significant differences in diagnostic or therapeutic delays were observed based on sex, age (≤65 vs >65 years), Karnofsky Performance Status (KPS ≥ 70% vs <70%), or involvement of deep brain structures. Geographic location, including residence in a rural area and therefore greater distance from a maximum care hospital, did not significantly impact diagnostic or therapeutic timelines. Patients presenting with cognitive deficits as the primary symptom did not experience significantly longer delays in diagnosis compared to those with more distinct clinical presentations, such as focal neurological deficits or epileptic seizures (Table 2).

### Treatment and Survival Analyses

**Figure 1.**
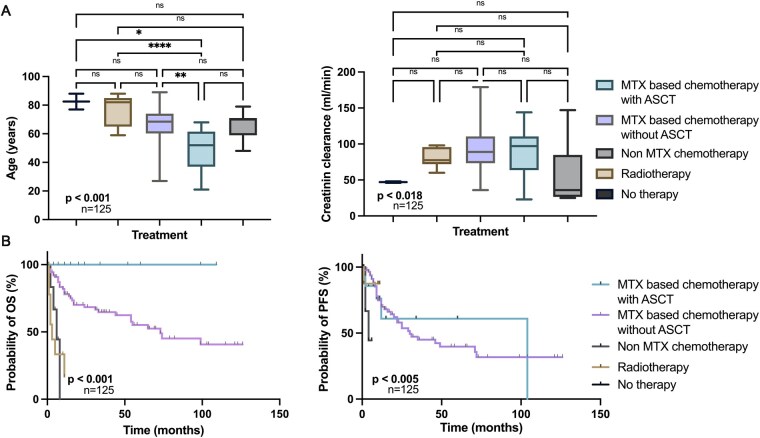
Subgroup differences of age, renal function and outcome according to different treatment regimens. Subgroup analysis illustrates age and renal function (creatinine clearance ml/min) distribution among the treatment groups (A). Patients who received palliative treatment (no therapy or radiotherapy) were the oldest and had the worst renal function. The patients treated with MTX with ASCT had a median age of 52 years, which was significantly different from most other treatment groups (A). Kaplan Meier survival estimates of OS and PFS according to treatment (B). OS and PFS differed significantly among the treatment groups (*P* < .001, *P* < .005). Asterisks indicate the level of statistical significance, with a greater number of asterisks corresponding to lower *P*-values (*P < .05; **P < .01; ***P < .001; ****P < .0001).

The median OS in the entire cohort was 73 months (95% CI: 46.86-99.14). The majority of patients (72.8%) received methotrexate (MTX)-based polychemotherapy without autologous stem cell transplantation (ASCT), while 11.2% received MTX-based polychemotherapy with ASCT. A smaller proportion (5.6%) underwent non-MTX-based polychemotherapy, 8.8% received radiotherapy alone, and 1.6% received best supportive care. Patients undergoing MTX and ASCT had the lowest median age (52 years, range: 21-68), which was significantly different compared to other treatment groups (*P* < .001, Figure 1A). They also had the highest median KPS (80%, range: 60-100) (Table 3). Renal function was also superior in this group, with a median creatinine clearance of 97 mL/min (range: 23-144) (Figure 1). As expected, patients in the non-MTX chemotherapy group exhibited the worst renal function, with a median creatinine clearance of 36 mL/min (range: 25-147) (Table 3 and Figure 1). OS and PFS differed significantly among treatment groups (*P* < .001, *P* < .005, respectively, Figure 1).

**Table 3.** Overall survival and progression free survival according to therapy regimens. Median Age, KPS and renal function (creatinine clearance) in the different therapy subgroups

	*N* = 125 (%)	Overall survival (months); median (95% CI)	Progression free survival (months); median (95% CI)	Age (years); median (range)	KPS (%); median (range)	Creatinine clearance (ml/min)
MTX-based polychemotherapy with ASCT	14 (11.2)	Not reached*	104 (31.33-101.25)	52 (21-68)	80(60-100)	97 (23-144)
MTX-based polychemotherapy without ASCT	91 (72.8)	73 (65.211-97.669)	30 (43.502-72.002)	68 (27-86)	70(20-100)	88 (36-179)
Non-MTX based polychemotherapy without ASCT	7 (5.6)	6 (3.763-8.015)	4 (2.778-5.667)	70 (48-79)	70 (40-80)	36 (25-147)
Radiotherapy	11 (8.8)	3 (1.895-7.600)	**	82 (59-88)	70 (50-90)	77.5 (60-98)
Best supportive care	2 (1.6)	0	***	82.5 (77-88)	15 (10-20)	47 (46-48)

* No deaths were reported in this group during a median follow up of 104 months.

** In the radiotherapy group, only one patient lived long enough, so that progression was noted. Therefore, no median PFS can be calculated.

*** Progression free survival could not be calculated as all patients who received best supportive care died within 2 months.

Patients receiving MTX-based chemotherapy with ASCT exhibited the longest OS, with no deaths reported (Table 3). Therefore, OS in the MTX+ASCT group was significantly longer than in those receiving MTX-based chemotherapy alone (median OS of 73 months). PFS was longer in the MTX+ASCT group (104 months; 95% CI: 31.33-101.25), whereas in the MTX-only group median PFS was 30 months (95% CI: 43.502-72.002). Among patients receiving MTX-based chemotherapy without ASCT, median OS was significantly longer compared to those treated with non-MTX-based chemotherapy (73 vs 6 months, *P* < .001). Patients treated with radiotherapy alone had a median OS of 3 months, while those receiving best supportive care (*n* = 2) had a survival duration of less than one month (Table 3).

### Effects of Age, Diagnostic and Therapeutic Delay on Survival

**Figure 2.**
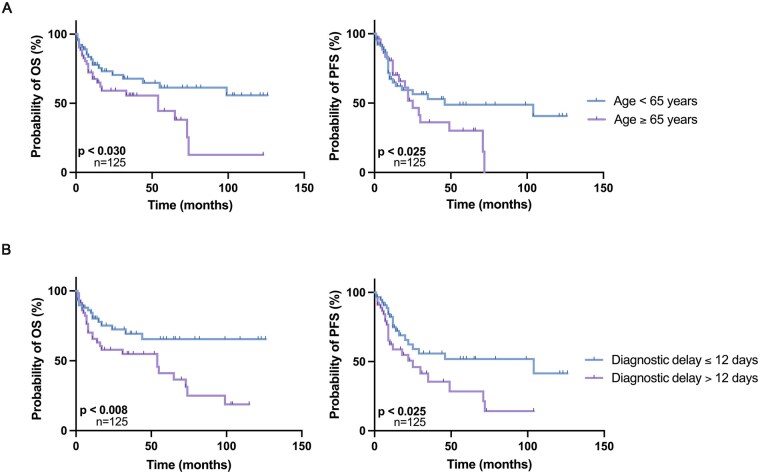
Kaplan-Meier survival estimates of OS and PFS according to age and diagnostic delay. Kaplan Meier survival estimates of overall survival (OS) and progression free survival (PFS) according to age and diagnostic delay. Patients aged younger than 65 years survived significantly longer (*P* < .030), and PFS was prolonged in younger patients (*P* < .025). Also diagnostic delay more than 12 days (calculated with the time from first neuroimaging until histopathological diagnosis) was significantly associated with inferior OS and PFS (*P* < .008, *P* < .025, respectively).

Patients with a diagnostic interval (time from first neuroimaging to histopathological diagnosis) shorter than the median of 12 days had significantly longer OS (149 vs 54 months, log-rank test with *P* < .008, Figure 2B). PFS was also significantly extended in patients with shorter diagnostic delay (15 vs 11 months, median: 14 months, log-rank test with *P* < .025, Figure 2B). OS was not significantly influenced by the time from initial symptom onset to histopathological diagnosis, in PFS there was a trend towards a shorter PFS. Neither OS nor PFS was significantly influenced by the time from initial symptom onset to histopathological diagnosis (*P* < .596 and *P* < .169, respectively), the time from neuroimaging to treatment (*P* < .11, *P* < .289), or the time from diagnosis to treatment initiation (*P* < .280 for OS, *P* < .149 for PFS) (Supplementary Table S1). In Kaplan-Meier analysis we also found significant superior OS and PFS in younger patients (<65 years) (*P* < .030, *P* < .025 respectively, Figure 2A).

### Univariate Analysis Based on a Median Split of Time from Neuroimaging to Treatment

#### Prognostic factors

##### Overall survival

Univariate analysis identified several significant prognostic factors for OS, including age <65 years, KPS, pre-biopsy immunosuppressive medication, renal function (glomerular filtration rate, GFR), diagnostic delay (time from first neuroimaging to histopathological diagnosis), and treatment modality, particularly MTX-based chemotherapy (Supplementary Table S1). However, the time from diagnosis to treatment initiation did not significantly impact OS (*P* < .280, median time 7 days [0-81]). Patients with a higher tumor burden (>6.9 cm^2^) tended to have poorer OS compared to those with a lower burden (≤6.9 cm^2^), though this difference did not reach statistical significance (*P* < .055).Variables such as sex, deep brain structure involvement, and CSF protein elevation—previously reported as ­prognostic markers—were not significant in our cohort ­(Supplementary Table S1).

Multivariate Cox regression analysis identified KPS as an independent predictor of OS (*P* < .003), with an odds ratio (OR) of 0.370, indicating that higher KPS (>70%) was associated with a lower mortality. Renal function also had a significant impact on OS (*P* < .027, OR = 0.483), suggesting a protective role for preserved renal function.

Treatment modality significantly influenced OS outcomes. Using MTX-based chemotherapy as the reference group, patients receiving best supportive care, radiotherapy alone, or non-MTX chemotherapy had a significantly increased mortality (*P* < .001 and *P* < .046, respectively). In contrast, age, diagnostic delay, and pre-biopsy immunosuppression were not independently associated with OS in the multivariate model (Table 4).

**Table 4.** Predictors of overall survival (OS) and progression free survival (PFS) in the multivariate Cox proportional hazards model

*Clinical variable*	*Hazard ratio (P-value)*	*Hazard ratio (P-value)*
	*OS*	*PFS*
*Age <65 years*	*1.096 (P = .777)*	*Not included*
*Age ≥ 65 years*		
*KPS ≥ 70%*	*0.370 (P* < .**003** *)*	*Not included*
*KPS < 70%*		
Drug immunosuppression 14 days prior to biopsy	*0.786 (0.491)*	*Not included*
No immunosuppressants		
*Localization*	*Not included*	*P* = ***0.41***
*Supratentorial only*		*0.067 (P* = .***012)***
*Infratentorial only*		*0.112 (P* = .*050)*
*Supra- and infratentorial*		*0.156 (P* = .*086)*
*Spinal*		*0.000 (P = .975)*
*Time from first ­neuroimaging until histopathological diagnosis*	*1.498 (P = .221)*	*2.028* ***(*** *P* = .***024)***
*≤ 12 days*		
*> 12 days*		
*Renal function*	*0.483 (P* = .***027*** *)*	*0.581 (P = .341)*
*GFR < 60 mL/min*		
*GFR ≥ 60 mL/min*		
*MTX-based chemotherapy*	*P* < .***001***	*P = .126*
*MTX + ASCT*	*0.000 (P = .961)*	*1.*589 (*P* = .356)
*Non-MTX*	*3.461 (P* = .***046)***	*5.829 (P* = .***023*** *)*
*No therapy/radiotherapy*	*6.902 (P* < .***001*** *)*	*1.507 (P = .695)*

Bold values indicate statistical significance.

##### Progression-free survival

Univariate analysis identified tumor localization, diagnostic delay, and treatment modality as significant predictors of PFS. Renal function was also included in the multivariate model due to a near-significant association (*P* < .061).

Multivariate analysis demonstrated that diagnostic delay was significantly associated with unfavourable PFS (*P* < .024, OR = 2.028), indicating that longer diagnostic intervals were linked to an increased risk of disease progression. While treatment modality in general did not show an overall significant effect (*P* < .126), subgroup analysis revealed that patients receiving non-MTX chemotherapy had a significantly higher risk of progression (*P* < .023, OR = 5.829).

Tumor localization also emerged as a significant predictor of PFS. Specifically, supratentorial tumor localization was associated with a reduced risk of progression (*P* < .012, OR = 0.067).

#### Immunosuppressant exposure

We performed an additional subgroup analysis comparing patients with prior immunosuppression (predominantly corticosteroids, *n* = 48) to those without (*n* = 77) (Supplementary Table S2). Immunosuppressed patients presented with significantly smaller SPD at diagnosis (5.6 cm^2^ compared to 7.5 cm^2^, *P* = .36), consistent with the known cytoreductive effect of steroids. KPS was not significantly different in the two groups. Moreover, diagnostic intervals from symptom onset to histopathological confirmation as well as from first neuroimaging to treatment initiation were significantly prolonged in the immunosuppressed group (*P* = .04, *P* < .001 respectively).

## Discussion

In this retrospective, monocentric study, we evaluated the impact of diagnostic delay and key clinical timelines on survival outcomes in patients with PCNSL. Our findings highlight the importance of a rapid diagnostic process, particularly the time from first neuroimaging to histopathological confirmation, as a crucial determinant of PFS. In summary, our key findings were as follows:

First, a shorter diagnostic interval (≤12 days from first neuroimaging to histopathological confirmation) was significantly associated with longer OS and PFS, with multivariate analysis confirming diagnostic delay as an independent prognostic factor for PFS. Second, among the possible factors contributing to diagnostic delay, prebiopsy immunosuppressive therapy emerged as a significant determinant, while initial misinterpretation of neuroimaging findings exhibited a trend toward prolonged time to diagnosis. Third, multivariate analysis identified KPS and renal function as independent predictors of OS, indicating that general functional status and adequate renal function are crucial determinants of survival. Also, supratentorial tumor localization was associated with a lower risk of progression. Fourth, treatment with MTX-based chemotherapy, particularly in combination with ASCT, was associated with the longest OS and PFS and patients treated with MTX-based chemotherapy had significantly better survival compared to those receiving non-MTX regimens, radiotherapy alone, or best supportive care.

To the best of our knowledge this is the first study reporting diagnostic delay as an independent prognostic factor for PFS in PCNSL. These findings contrast with prior studies, which did not identify a significant correlation between diagnostic delay and survival.^10^^,^^17^ Importantly, although diagnostic delay in our cohort showed considerable variability, the overall duration was shorter than in previously reported series (median time from first neuroimaging to histopathological confirmation: 12 days). This shorter diagnostic interval may help explain the observed survival benefit, as it was followed by treatment initiation that occurred after extremely short median delays (7 days) with lower overall variability.^18^^,^^19^ Therefore, the potentially modifiable variable in real-world clinical practice is the diagnostic delay, not the treatment delay once a diagnosis has been made. The median time from symptom onset to histopathological diagnosis did not significantly impact survival (37 days in our cohort vs 47-76 days in recent studies), which may be due to recall bias, as early symptoms of PCNSL (such as cognitive or behavioral changes) are often nonspecific and challenging to accurately pinpoint. Similarly, the interval from histopathological confirmation to treatment initiation was shorter in our cohort (7 vs 20 days in previous studies), but did not have a significant effect on OS or PFS.^10^ However, given the already short median interval in our study, statistical power may be limited in detecting further benefits from expediting treatment initiation. Collectively, our results emphasize that the critical window influencing survival in PCNSL lies between initial neuroimaging and histopathological confirmation. Therefore, streamlining the diagnostic workup, reducing misdiagnosis, and expediting biopsy procedures may yield significant clinical benefits.^16^ Given these considerations, diagnostic delay may be regarded as an exploratory prognostic variable of potential relevance for clinical practice; however, its impact should be interpreted with caution within the multifactorial real-world setting.

Thus, we tried to identify crucial modifiable factors that may contribute to diagnostic delay. In our cohort, two contributors emerged: Patients receiving immunosuppressive treatment in the 14 days preceding biopsy experienced significantly longer diagnostic delays, despite all cases being histologically confirmed on the first biopsy attempt. This phenomenon is well-documented, as described by Baraniskin et al and Bromberg et al, who report that immunosuppression may obscure or alter the radiological and clinical presentation of PCNSL, leading to delayed diagnosis.^20^ Given the high sensitivity of lymphoma cells to corticosteroid-induced apoptosis, corticosteroid administration can distort tumor morphology and, in some cases, even result in tumor disappearance.^21^ In our subgroup analysis of immunosuppressant exposure, prior immunosuppression was associated with smaller radiographic tumor burden and worse functional status, as indicated by lower KPS scores. In the univariable analysis, prior intake of immunosuppressive medication was significantly associated with shorter OS. However, this effect did not remain significant in the multivariable model, where performance status (KPS), treatment, and renal function demonstrated stronger independent associations with survival. This suggests that while immunosuppressive medication may influence OS, its effect is likely mediated or outweighed by other prognostic factors.

Although not statistically significant, a trend toward longer diagnostic intervals was observed when the initial radiological assessment did not suggest lymphoma. Radiological key features as supratentorial, periventricular, multilocular localization (often crossing the midline) with restricted diffusion and contrast enhancement do not always apply.^22^ This underscores the need for heightened radiological awareness of PCNSL’s varied imaging presentation to minimize delays. Importantly, system-related rather than patient-related factors appeared to influence diagnostic and therapeutic timelines. Neither geographic location (metropolitan vs rural areas), age, sex, KPS, nor deep brain involvement significantly impacted the diagnostic process. These findings suggest that institutional protocols, radiological interpretation, and early clinical suspicion may play a more decisive role in expediting diagnosis than individual patient characteristics.

Multivariate analysis confirmed KPS, treatment modality and renal function as independent prognostic factors for OS. While age has traditionally been included in established prognostic models such as the IELSG and MSKCC scores, it did not emerge as an independent predictor in our cohort.^14^^,^^15^ In contrast, renal function (GFR) was a significant determinant of survival, a finding not previously emphasized in PCNSL prognosis. Preserved renal function is essential for the administration of high-dose methotrexate (MTX), the cornerstone of PCNSL treatment. Given that MTX clearance depends on renal function, it is plausible that patients with better renal function tolerate higher MTX dosages and therefore achieve superior treatment outcomes. While creatinine clearance was only significant in univariate analysis in prior studies, our findings indicate that renal function independently predicts OS, irrespective of treatment modality.^15^ Similar prognostic implications of renal function have been observed in other malignancies, including breast, ovarian, colorectal, lung, and prostate cancers.^23–25^ Interestingly, tumor localization emerged as a significant predictor of PFS, with supratentorial involvement associated with a lower risk of progression. This may be attributed to greater disruption of the blood-brain barrier in the brain parenchyma, potentially enhancing chemotherapy efficacy.^26^ The impact of tumor localization on survival was also demonstrated in PCNSL by Lin et al,^27^ who reported poorer outcomes in patients with basal ganglia involvement.

Our findings reinforce the central role of MTX-based chemotherapy as the standard of care and support the use of ASCT in eligible patients. Ultimately it is not surprising that chemotherapy compared to radiotherapy or best supportive care confirmed to be an independent prognostic factor of OS. OS and PFS were significantly longer in patients who received MTX-chemotherapy and ASCT. This is in line with a large meta-analysis of clinical trials in PCNSL, which also showed that chemotherapy and ASCT were associated with the best survival rates.^28^ A recently conducted Phase III trial, comparing nonmyeloablative chemoimmunotherapy compared to high dose chemotherapy followed by ASCT also showed significantly better outcomes in the ASCT group.^12^ Despite its superior efficacy, ASCT remains feasible only for a select subgroup of patients, primarily younger individuals with good performance status. In our cohort, ASCT recipients had a median age of 52 years and a median KPS of 80%, comparable to prior studies where eligibility criteria were stringent.^12^ Given that KPS itself was an independent prognostic factor, it is possible that the better outcomes in the ASCT group partly reflect superior baseline functional status rather than the treatment effect alone. Our findings support the beneficial role of ASCT in eligible patients, in line with previous reports. However, only a minority of patients in our cohort were treated with this modality, reflecting both the restrictive eligibility criteria and the real-world limitations of this therapeutic approach.

In summary, our study underscores the importance of early and accurate diagnosis in PCNSL. Given the aggressive nature of the disease, optimizing diagnostic pathways should be a priority. Standardized protocols, including streamlined access to stereotactic biopsy and reduced use of empiric steroid treatment before histological confirmation, could improve diagnostic delays. One might speculate whether diagnostic delay could be reduced through the implementation of rapid, and noninvasive diagnostic approaches, such as the detection of circulating tumor DNA or other molecular diagnostics, thereby enhancing detection rates and potentially improving long-term outcomes.^29–31^ Additionally, increased clinical awareness of PCNSL's diverse radiological presentation is crucial to avoid misdiagnosis and prevent unnecessary delays. While acknowledging the limitation of our small sample size, from a therapeutic perspective, our findings reinforce MTX-based chemotherapy as the best treatment strategy, particularly in combination with ASCT in eligible patients. Furthermore, given the prognostic importance of renal function, nephrotoxicity monitoring and optimization of renal function before initiating MTX therapy should be emphasized.

Despite the insights gained from our study several limitations need to be acknowledged. Although we analyzed a comparatively large cohort for a rare disease such as PCNSL, from a statistical perspective the sample size remains relatively small, carrying a risk of statistical error due to the limited number of patients available for some analyses. Also the retrospective design and single-center setting may impact generalizability. The study lacks important data as neuropsychological testing at first diagnosis, therapy associated toxicity and neurotoxicity, as detailed information was not available. As pointed out diagnostic delay was not modulated from patient-derived factors, but system-related factors, and comparison with other centers and standard-operating-procedures would be interesting in comparing outcome. Additionally, while our data suggest that reducing diagnostic delay improves outcomes, prospective studies are needed to determine whether specific interventions, such as rapid-access diagnostic pathways, can significantly improve survival. Further research should also focus on identifying additional modifiable factors contributing to diagnostic delay, as well as investigating the impact of novel therapeutic strategies on survival in PCNSL.

## Conclusion

This study suggests that in PCNSL, a shorter interval between first neuroimaging and histopathological diagnosis may be associated with longer PFS, underscoring the potential prognostic relevance of diagnostic delay. We also identify renal function and KPS as independent prognostic markers of OS. Our findings reinforce MTX-based chemotherapy as the standard of care, with ASCT offering the best survival outcomes in eligible patients. Given that diagnostic delay is a modifiable factor, promoting clinical awareness and implementing standardized diagnostic protocols could have a meaningful impact on patient outcomes.

## Supplementary Material

vdaf234_Supplementary_Data

## Data Availability

The data will be made available upon reasonable request.
