# Supplementary material for: Time matters: The prognostic impact of diagnostic delay on survival in primary central nervous system lymphoma—a single-center, retrospective real-world study
Source: Neurooncol Adv. 2025 Oct 27;8(1):vdaf234. doi: 10.1093/noajnl/vdaf234 (PMC12901676; doi:10.1093/noajnl/vdaf234)
Supplement: vdaf234_Supplementary_Data [file vdaf234_supplementary_data.zip › Supplementary material revision-clean.docx]

**Supplementary material**

Suppl. Table 1 Predictors of overall survival (OS) and progression free survival (PFS) in the univariate analysis

|  | Univariate analysis OS | | Univariate analysis PFS | |
| --- | --- | --- | --- | --- |
|  | N | p-value | N | p-value |
| Age <65 years  Age ≥ 65 years | 64  59 | **0.008** | 63  59 | 0.340 |
| *Male*  Female | 73  50 | 0.784 | 73  49 | 0.887 |
| *KPS ≥ 70%*  KPS < 70% | 78  45 | **<0.001** | 45  77 | 0.915 |
| *Ki67 ≤ 70%*  Ki67 >70% | 46  74 | 0.604 | 46  73 | 0.659 |
| Drug immunosuppression 14 days prior to biopsy  No immunosuppressants | 48  77 | **0.017** | 48  77 | 0.992 |
| *No involvement of deep brain structures*  Involvement of deep brain structures | 33  90 | 0.928 | 33  89 | 0.402 |
| *Singular lesion*  *Multiple lesions* | 47  75 | 0.702 | 46  75 | 0.090 |
| *Tumor area (SPD) (cm^2^)*  *≤ 6.9*  *> 6.9* | 62  63 | *0.055* | 62  63 | *0.238* |
| *Localisation*  *Supratentorial only*  *Infratentorial only*  *Supra- and infratentorial*  *Spinal* | 77  17  26  1 | 0.215 | 77  16  26  1 | **0.044** |
| *Ocular involvement*  *No ocular involvement* | 7  116 | 0.362 | 7  115 | 0.248 |
| *Normal CSF cell count*  *Elevated CSF cell count* | 50  61 | 0.372 | 50  60 | 0.355 |
| *Normal CSF protein*  *Elevated CSF protein* | 14  89 | 0.944 | 14  88 | 0.673 |
| *Main symptom*  *Cognitive impairment*  *Other neurological deficits* | 44  79 | 0.100 | 44  78 | 0.089 |
| *Leading symptom*  *Cognitive deficits*  *Epileptic seizure*  *Focal neurological deficits (sensory, motor, visual)*  *Cerebellar symptoms*  *Aphasia* | 44  11  37  26  5 | 0.191 | 44  11  37  25  5 | 0.143 |
| *Metropolitan area*  *Rural area* | 43  80 | 0.192 | 79  43 | 0.714 |
| *Initial radiological assessment*  *CNS lymphoma*  *Other differential diagnoses* | 85  38 | 0.599 | 85  37 | 0.095 |
| *Renal function*  *GFR < 60 ml/min*  *GFR ≥ 60 ml/min* | 19  104 | **<0.001** | 19  103 | 0.061 |
| *Time from first neuroimaging until histopathological diagnosis ≤ 12 days*  *> 12 days* | 65  61 | **0.018** | 65  57 | **0.024** |
| Time from initial symptom until histopathological diagnosis  *≤ 37 days*  *> 37 days* | 66  60 | 0.596 | 16  106 | 0.169 |
| Time from histopathological diagnosis to treatment  *≤ 7 days*  *> 7 days* | 68  58 | 0.280 | 64  58 | 0.149 |
| Time from neuroimaging to treatment  *≤ 20*  > 20 | 66  59 | 0.110 | 66  59 | 0.289 |
| *Best supportive care*  *Radiotherapy* | 2  11 | **0.049** | 2  11 | censored |
| *No therapy/Radiotherapy*  *MTX-based C*  *MTX + ASCT*  *Non-MTX* | 13  88  14  7 | **0.008** | 13  88  14  7 | 0.079 |
| *MTX-chemotherapy*  *Non-MTX chemotherapy* | 89  7 | **0.003** | 88  7 | **0.001** |
| *MTX-chemotherapy without ASCT*  *MTX-chemotherapy with ASCT* | 89  14 | 0.145 | 88  14 | 0.928 |

*Suppl. Table 2: Subgroup analysis of immunosuppressed and not immunosuppressed patients 14 days prior to biopsy*

|  | *No immunosuppression*  *(N =77)* | *Immunosuppression 14 days prior to biopsy*  *(N=48)* |  |
| --- | --- | --- | --- |
|  | *Median (range)* | | *p-value* |
| *Tumor area (SPD) (cm2)* | *7.5 (0-39)* | *5.6 (0-51)* | ***0.036*** |
| *KPS (%)* | *80 (10-100)* | *70 (40-90)* | *0.062* |
| *Time from initial symptom until histopathological diagnosis* | *32 (4-506)* | *45 (10-749)* | ***0.004*** |
| *Time from first neuroimaging until histopathological diagnosis* | *9 (2-110)* | *18 (2-225)* | ***< 0.001*** |
| *Time from histopathological diagnosis to treatment* | *6 (0-81)* | *8 (0-31)* | *0.074* |
| *Time from neuroimaging to therapy initiation* | *16 (4-114)* | *26 (4-228)* | ***< 0.001*** |

*p-value was calculated using Mann-Whitney-U-Test*

*
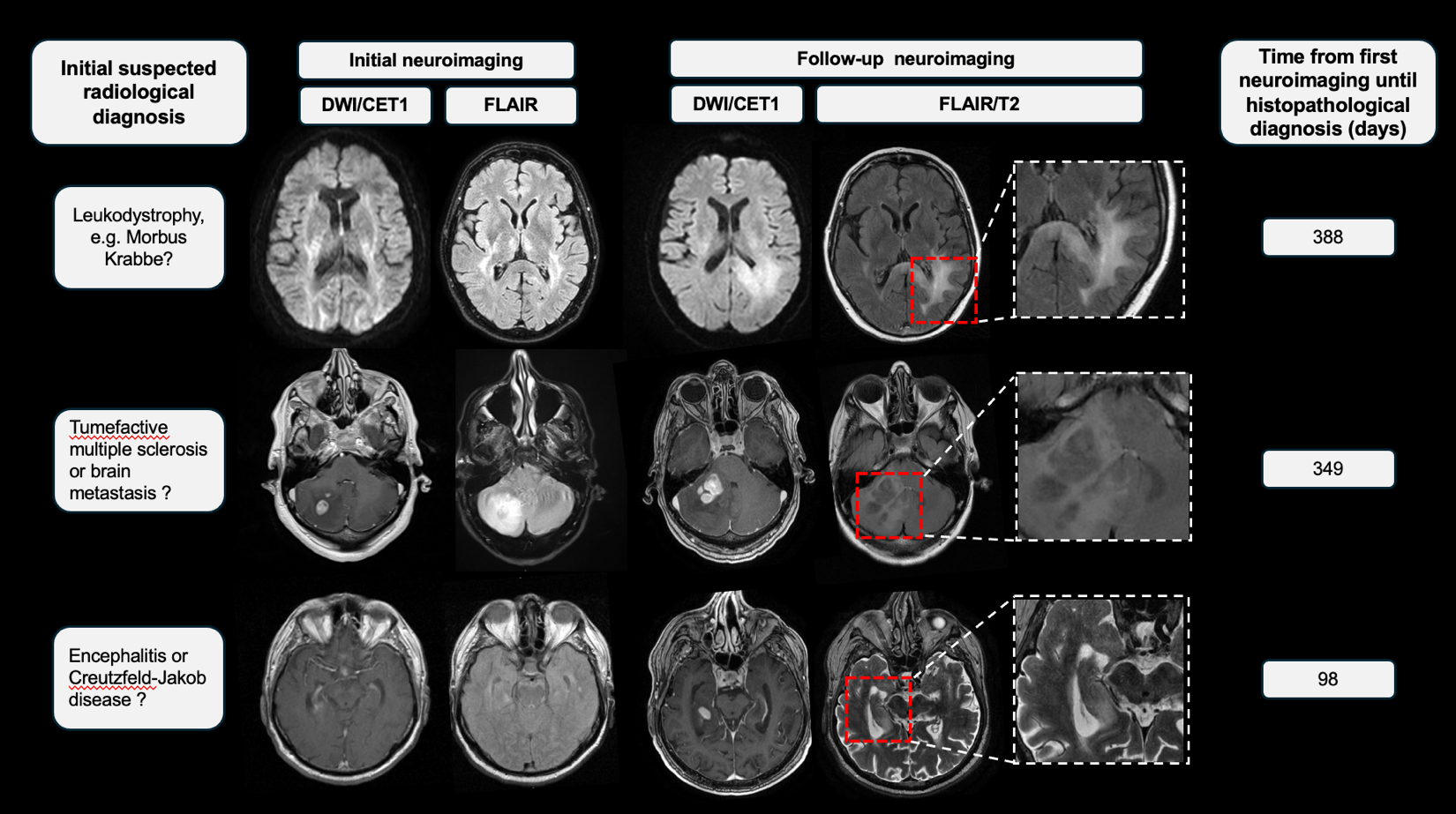
Suppl. Figure 1 Representative Examples of initial misdiagnosis on neuroimaging and cases with primary central nervous system lymphoma mimics. Time from first neuroimaging until histopathological diagnosis is given; DWI: diffusion weighted imaging, FLAIR: Fluid-attenuated inversion recovery, CET1: Contrast-enhanced T1 weighted*
